# [D-Ala^2^, D-Leu^5^] Enkephalin Inhibits TLR4/NF-*κ*B Signaling Pathway and Protects Rat Brains against Focal Ischemia-Reperfusion Injury

**DOI:** 10.1155/2021/6661620

**Published:** 2021-02-12

**Authors:** Danyun Fu, Haitong Liu, Jiang Zhu, Hongjiao Xu, Junyan Yao

**Affiliations:** ^1^Department of Anesthesiology, Shanghai General Hospital, Shanghai Jiao Tong University School of Medicine, No. 100, Haining road, Hongkou district, Shanghai 200080, China; ^2^Department of Anesthesiology, the Second Affiliated Hospital of Soochow University, Suzhou 215006, China

## Abstract

**Background:**

Cerebral ischemia-reperfusion (I/R) injury is the main cause of acute brain injury, which is a life-threatening disease due to the lack of effective treatments. [D-Ala^2^, D-Leu^5^] enkephalin (DADLE) is a synthetic delta-opioid receptor agonist that is reported to confer neuroprotective effect; however, the underlying mechanism is still being explored. The purpose of the present study is to determine whether DADLE administrated intracerebroventricularly could attenuate the cerebral I/R injury, to determine if this is through inhibiting the toll-like receptor 4 (TLR4)/nuclear factor kappa B (NF-*κ*B) signaling pathway and therefore inhibiting neuroinflammation in an ischemic stroke model.

**Methods:**

Rats were subjected to 120 minutes of ischemia by transient middle cerebral artery occlusion (MCAO). At 45 minutes after ischemia, DADLE or control vehicle (artificial cerebrospinal fluid, ACSF) was given to the rats intracerebroventricularly. Neurological deficit, cerebral infarct volume, and histopathological changes were assessed at 24 hours after reperfusion. Brain inflammation was assessed by measuring tumor necrosis factor-*α* (TNF-*α*) and interleukin-6 (IL-6) in the ischemic penumbra by ELISA. The expression of TLR4 was determined by immunohistochemistry staining and western blotting. The expression of NF-*κ*B was investigated by western blotting.

**Results:**

Compared with the vehicle-treatment (ACSF), DADEL improved neurological deficit (9.6 ± 2.1 versus 13.8 ± 1.9), reduced cerebral infarct volume (18.74 ± 3.30% versus 10.57 ± 2.50%), and increased the number of normal neurons (29.72 ± 8.53% versus 51.37 ± 9.18%) after cerebral I/R injury in rats (all *P* < 0.05). Expressions of inflammatory molecules including TNF-*α* and IL-6 were highly expressed in the vehicle-treated rats, whereas treatment with DADLE downregulated these expressions (*P* < 0.05). Additionally, cerebral I/R injury significantly increased the TLR4 and NF-*κ*B expression in vehicle-control group, which was markedly inhibited by DADLE (*P* < 0.05).

**Conclusions:**

DADLE, administrated intracerebroventricularly at 45 minutes after cerebral ischemia, significantly ameliorated I/R-induced brain damage in rats. This kind of neuroprotective effect appears to be related to the downregulation of TLR4-mediated inflammatory responses.

## 1. Introduction

Stroke is one of the most life-threatening diseases leading to significant mortality and long-lasting disability [1]. Thrombolysis with tissue-type plasminogen activator is an effective treatment for acute ischemic stroke; however, its clinical application is limited by the short treatment window and risk of hemorrhage [2]. Therefore, it is imperative to develop novel therapeutic pharmacological agents for stroke therapies.

[D-Ala^2^, D-Leu^5^] enkephalin (DADLE) is a synthetic delta-opioid receptor agonist which has been shown to execute neuroprotection against ischemia. Our previous study demonstrated that infusion of DADLE could alleviate spinal cord I/R injury in a rabbit model [3]. Previous experimental studies in rat found that DADLE could significantly reduce hippocampal CA1 neuronal loss and attenuate neurological impairments following global cerebral ischemia caused by hypotension or cardiac arrest [4, 5]. However, the mechanism of DADLE-induced neuroprotection is still being explored.

It involves highly complex mechanisms concerning cerebral ischemia-reperfusion (I/R) injury [6, 7]. Therein, inflammatory response is involved in acute ischemic stroke pathophysiology and constitutes a potential therapeutic target for the development of neuroprotective drugs [8–10]. In previous experimental studies, DADLE showed neuroprotective effects, including antioxidant and antiapoptotic effects [11]. However, whether DADLE could alleviate neuronal damage by inhibiting inflammatory factors in the central nervous system is not yet clear.

Toll-like receptors (TLRs) are types of pattern recognition receptors in innate immunity that respond to systemic infectious or noninfectious inflammatory reactions. Among the family of TLRs, toll-like receptor 4 (TLR4) plays an important role during the pathological process of cerebral I/R injury [12]. Previous studies suggested that the TLR4 knockout mice had significantly less cerebral infarct volume and improved neurological impairment produced by cerebral ischemia compared with the wild-type mice [13–15]. Furthermore, it has been shown that TLR4 activating generally induced the expression of nuclear factor kappa B (NF-*κ*B) and inflammatory cytokines, including tumor necrosis factor-*α* (TNF-*α*) and interleukin-6 (IL-6), and finally triggered intense inflammatory signaling and aggravated the brain damage [16].

Using a transient MCAO stroke model in rats, we firstly aimed to verify DADLE would reduce focal cerebral I/R injury. Secondly, we aimed to test the protective effect of DADLE related to the suppression of neuroinflammation. Furthermore, we explored that the anti-inflammatory effect by DADLE was associated with the downregulation of TLR4/NF-*κ*B signaling pathway.

## 2. Materials and Methods

### 2.1. Experimental Groups and MCAO Model

Adult male Sprague Dawley rats weighing 200-240 g were supplied by Shanghai Jiao Tong University. Animals were housed in a room under constant temperature (20-25°C) and a 12-h light/dark cycle, with food and water available. All experimental procedures were conducted according to the *Guide for the Care and Use of Laboratory Animals* and were approved by the Animal Ethics Committee of Shanghai General Hospital, Shanghai Jiao Tong University School of Medicine (IACUC: 2019AW027).

The MCAO model of transient focal I/R in rats was established as described in a previous study [17]. Briefly, animals were anesthetized by intraperitoneal injection of pentobarbital sodium at an initial dose of 40 mg/kg and at the maintained dose of 10 mg/kg during the experiment. The right femoral artery was cannulated for continuous monitoring of the mean arterial pressure (MAP). The right common carotid artery, external carotid artery, and internal carotid artery were carefully exposed and isolated. Then, the middle cerebral artery was occluded by inserting a 4-0 monofilament nylon suture (diameter 0.32 ± 0.02 mm; Beijing Cinontech Co., Ltd., Beijing, China) with a rounded tip from the common carotid artery into the internal carotid artery until a slight resistance was felt. The animals were allowed to recover from anesthesia after intracerebroventricular administration of drugs. The rectal temperature was monitored and maintained at 36.5 to 37.5°C with a warming blanket and heating lamp during the experiments. At 2 hours after ischemia, the suture was removed and reperfusion was performed for 24 hours. At the end of all experiments, the rats were euthanized by an overdose of anesthetic.

### 2.2. Experimental Design and Drug Disposition

**Figure 1:**
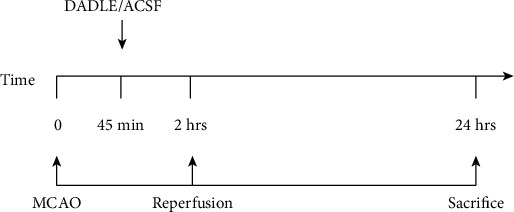
Time line of the experimental protocol. MCAO: middle cerebral artery occlusion; DADLE: [D-Ala^2^, D-Leu^5^]; ACSF: artificial cerebrospinal fluid.

The rats were randomly divided into the following three groups: Sham group (*n* = 15), artificial cerebrospinal fluid (ACSF) (vehicle) group (*n* = 18), and DADLE group (*n* = 18). The rats in the ACSF and DADLE groups were all subjected to ischemia by MCAO and then treated with ACSF or DADLE, respectively, at 45 minutes after cerebral ischemia via intracerebroventricular injection. While animals in the Sham group underwent operation without filament insertion. Time lines of the experimental protocol are presented in Figure 1.

DADLE (Sigma-Aldrich, St. Louis, MO, USA) 2.5 nmol was dissolved in 5 *μ*L ACSF (pH = 7.4, concentration mmol/L: NaCl 119, KCl 2.5, MgCl_2_ 1.3, KH_2_PO_4_ 1.0, CaCl_2_ 2.5, NaHCO_3_ 26.2, 12-glucose 11). This dose was based on previous reports combined with our preliminary studies [18]. The intracerebroventricular injection site is located at the following coordinates: 0.8 mm posterior to bregma, 1.5 mm lateral to the midline, and 3.8 mm beneath on the skull surface.

### 2.3. Neurological Deficit Evaluation and Infarct Volume Measurement

Before surgery and at 24 hours after reperfusion, an 18-point scale reported by Garcia et al. [19] was used to assess neurological deficit, including 6 items: 0-3 points, spontaneous activity; 0-3 points, symmetry of limb; 0-3 points, symmetry of forepaw outstretching; 1-3 points, climbing wire cage; 1-3 points, reaction to touch the left side of the body; and 1-3 points, response to touch the left side of vibrissae. Lower scores indicated a more severe neurological deficit. The neurological test was carried out by an examiner who was blinded to the experimental groups.

After neurologic deficit assessment, rats were anesthetized in a similar manner as described above. The brains were rapidly removed and sectioned coronally into six 2 mm slices for infarct volume analysis. Coronal sections were incubated in 2% 2,3,5-triphenyltetrazolium chloride (TTC, Sigma-Aldrich, St. Louis, MO, USA) at 37°C for 15 minutes, then photographed with a camera. The infarct region presented white, whereas the noninfarct tissue appeared red. To compensate for the brain edema, cerebral infarct percentage was calculated according to the following formula: {[total infarct area − (ipsilateral hemisphere area − contralateral hemisphere area)]/(contralateral hemisphere area × 2)} × 100%.

### 2.4. Histological Analysis of Brain Damage

After the neurological assessment, the rats were perfused with 4% paraformaldehyde in phosphate-buffered saline under general anesthesia. Samples of the ischemic penumbral region were harvested and then were fixed and embedded in paraffin. The method of collecting ischemic penumbral regions was based on the study of Ashwal et al. [20]. After dehydration in graded ethanol, specimens were serially sliced into coronal sections (5 *μ*m thick) for hematoxylin-eosin (HE) or immunohistochemical staining. Three nonoverlapping horizons were gathered from each section stained with HE and at least 30 cells in each horizon were counted by an investigator who was blinded to groups (>90 neurons). The survival rate [(the number of total cell − the number of damaged cell)/the number of total cell] was calculated to indicate the level of tissue injury. The damaged neurons were counted on the basis of the following standard: cell swelling, vacuolization, presence of shrunken, and darkened nuclei [17].

### 2.5. ELISA Assay

At 24 hours after reperfusion, rats were anesthetized with sodium pentobarbital (40 mg/kg), and brains were quickly removed under deep anesthesia. Tissue samples of ischemic penumbra were frozen in liquid nitrogen and stored at −80°C for ELISA and western blotting analyses. Inflammatory cytokines (TNF-*α* and IL-6) were measured by using TNF-*α* or IL-6 commercial ELISA kits (Antigenix America, USA) according to the manufacturer's instructions. The values were expressed as ng/mg protein in the cytoplasm.

### 2.6. Immunohistochemical Analysis

The paraffin-embedded brain sections were treated with 3% H_2_O_2_ for 10 minutes to block the endogenous peroxidase activity and then incubated at 37°C for 1.5 h with a rabbit polyclonal antibody against TLR4 (1 : 100; Novus Biologicals). After washing three times with phosphate-buffered saline, the sections were incubated with biotinylated secondary antibody (Boster Biotechnology Co. Ltd, Wuhan, China). Finally, diaminobenzidine was used as a peroxidase substrate. The sections were observed under 400 times of light microscopy, and three nonoverlapping photomicrographs were captured from each section. The numbers of positively stained cells in each high power field (HPF) were counted and expressed as (^−^x ± s)/HPF.

### 2.7. Western Blotting Analysis

At 24 hours after reperfusion, protein extraction reagents (Beyotime Biotech. Co., China) were used for extraction of nuclear and cytosolic proteins of the tissue samples according to the manufacturer's instructions. Whole protein weighing 50 *μ*g was used for measurement of the content of TLR4. Nuclear protein weighing 15 *μ*g was used for evaluation of the content of the NF-*κ*B p65 subunit. Protein samples were separated on 10% SDS polyacrylamide gels and transferred to nitrocellulose membranes. The nonspecific binding sites were blocked with 5% nonfat dry milk Tris-buffer. The membranes were subsequently incubated overnight at 4°C with a rabbit polyclonal antibody against TLR4 (1 : 250; Novus Biologicals) or a rabbit monoclonal antibody against NF-*κ*B p65 (1 : 1000; Cell Signaling Technology), followed by incubation with horseradish peroxidase-conjugated secondary antibody (1 : 2,0000, Jackson, American). The protein bands were visualized by a chemiluminescence detection system and exposed on an X-ray film. The expressions of TLR4 and NF-*κ*B p65 were normalized to *β*-actin to correct the variations of different samples. The optical densities of protein bands were measured by an image analysis software (Image Pro Plus 6.0).

### 2.8. Statistical Analysis

All results were enumeration data and were expressed as mean ± SD. The overall significance of the data was examined by one-way analysis of variance (ANOVA) followed by Tukey's post hoc test using SPSS 19.0 (Chicago, IL, USA). *P* < 0.05 was considered statistically significant.

## 3. Results

### 3.1. Mortality and Physiological Parameters

No mortality was observed in the sham-operated rats. Five animals were dead and were excluded due to subarachnoid hemorrhage or respiratory depression during 24 hours after reperfusion. The mortality was 3/18 in the ACSF group and 2/18 in the DADLE group. There was no significant difference in mortality between the two operated groups. The MAP and body temperature were maintained in the normal range during the whole experimental period. There were no significant differences in body weight, rectal temperature, and MAP in the three experimental groups (Table 1).

### 3.2. DADLE Improved Neurological Deficits, Infarct Volume and Histological Damage in a Rat MCAO Model

**Figure 2:**
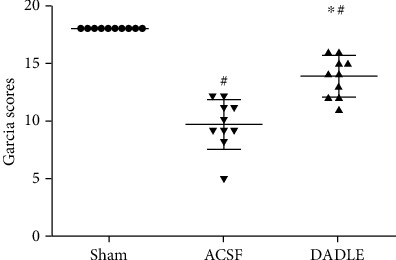
Neurologic deficit scores at 24 hours after reperfusion. DADLE treatment significantly improved the neurological scores. Bars represent mean ± SD (all groups *n* = 10). DADLE: [D-Ala^2^, D-Leu^5^]; ACSF: artificial cerebrospinal fluid. ^∗^*P* < 0.05 versus the ACSF group. ^#^*P* < 0.05 versus the Sham group.

**Figure 3:**
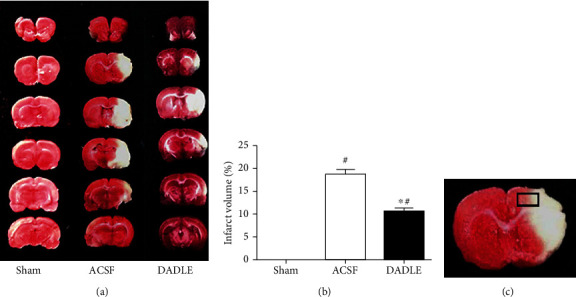
Cerebral infarct volume at 24 hours after reperfusion. (a) Brain slices after TTC staining from representative rats subjected to MCAO at 24 hours after reperfusion. The white area is infarct tissue, whereas red area is noninfarct region. (b) Percentage of brain infarct volume in the total brain. (c) Schematic representation of ischemic penumbral cortex taken for assay. Bars represent mean ± SD (all groups *n* = 5). DADLE: [D-Ala^2^, D-Leu^5^]; ACSF: artificial cerebrospinal fluid. ^∗^*P* < 0.05 versus the ACSF group. ^#^*P* < 0.05 versus the Sham group.

To determine whether DADLE had a neuroprotective property in a rat MCAO model, we firstly evaluated the neurological deficit, cerebral infarct volume percentage, and histopathological changes of the ischemic penumbral region after injection of DADLE, compared with ASCF. The neurological scores are shown in Figure 2. Compared with ASCF (9.6 ± 2.1), DADLE (13.8 ± 1.9, *P* < 0.05) significantly increased the average value of neurologic scores at 24 hours after MCAO (Figure 2). The images of continuous brain slices were shown in Figure 3(a), and accurate quantitative statistics of infarct volume were demonstrated in Figure 3(b). No cerebral infarction was observed in the sham-operated animals, suggesting that there was no brain damage due to the separation of the blood vessels. However, the infarct volume was 18.74 ± 3.30% of the total brain in the ACSF group, indicating that MCAO led to significant injury in the striatum and cortex. Compared with the ACSF group, DADLE treatment could significantly decrease the infarct volume to 10.57 ± 2.50% (*P* < 0.05) (Figure 3(b)). Then, the ischemic penumbral cortex was taken for follow-up study (Figure 3(c)).

**Figure 4:**
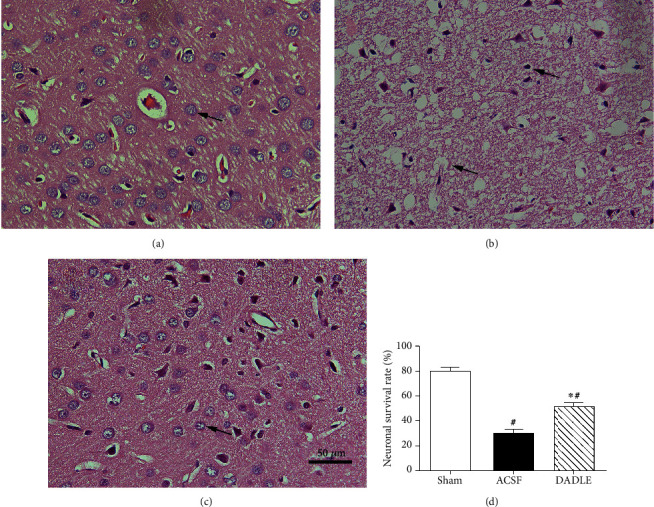
Histological features of the ischemic penumbral cortex at 24 hours after reperfusion. The Sham (a) group demonstrated abundance of normal neurons, while the ACSF (b) group showed significant reduction of viable neurons. Normal neuronal injuries were ameliorated in the DADLE group (c) (original magnification ×400). (d) Quantitative analysis of neuronal survival rate. Scale bar, 50 *μ*m. Bars represent mean ± SD (all groups *n* = 5). DADLE: [D-Ala^2^, D-Leu^5^]; ACSF: artificial cerebrospinal fluid. ^∗^*P* < 0.05 versus the ACSF group. ^#^*P* < 0.05 versus the Sham group.

HE staining revealed that the viable cells in the Sham group were arranged orderly, which had intact structure, prominent nucleolus, and abundant cytoplasm (Figure 4(a)). However, many neurons appeared shrunken and changed to vacuoles in the ACSF group (Figure 4(b)). This histological damage in the ischemic penumbra was ameliorated by DADLE (Figure 4(c)). The survival rate of neurons in the DADLE group (51.37 ± 9.18%) was significantly higher than that in the ACSF group (29.72 ± 8.53%, *P* < 0.05, Figure 4(d)).

### 3.3. DADLE Reduced Inflammatory Cytokine Levels in I/R-Induced Brain Injury

**Figure 5:**
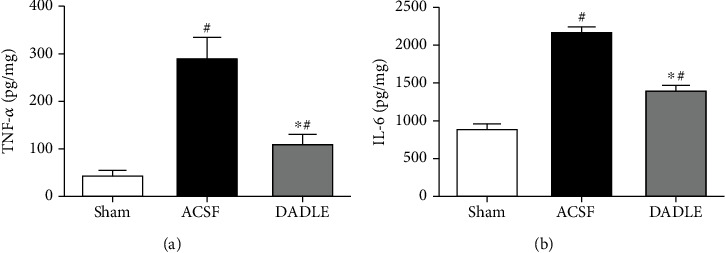
Suppression of I/R-induced TNF-*α* and IL-6 expressions by DADLE administration in the ischemic penumbra at 24 hours after reperfusion. Bars represent mean ± SD (group DADLE *n* = 6, other groups *n* = 5). DADLE: [D-Ala^2^, D-Leu^5^]; ACSF: artificial cerebrospinal fluid. ^∗^*P* < 0.05 versus the ACSF group. ^#^*P* < 0.05 versus the Sham group.

As reported, inflammatory responses play a crucial role in the pathophysiology of I/R brain damage. Inhibition of inflammatory reaction could attenuate the cerebral injury. To further evaluate the anti-inflammatory effect of DADLE, we examined the levels of TNF-*α* and IL-6 in the ischemic penumbra of the rat brain by ELISA. The content was at a low level in the brain of the Sham group. At 24 hours after reperfusion, the level of TNF-*α* showed a significant increase to 284.00 ± 100.32 pg/mL. DADLE treatment significantly attenuated these increases (63% of ACSF group, *P* < 0.05) (Figure 5(a)). In addition, the level of IL-6 was also increased significantly at 24 hours postoperatively in the ACSF group (2160.00 ± 140.41 pg/mL) compared with the Sham group (877.33 ± 176.00 pg/mg, *P* < 0.05). This increase in IL-6 was attenuated by DADLE treatment (36% of ACSF group, *P* < 0.05) (Figure 5(b)).

### 3.4. DADLE Inhibited the TLR4/NF-*κ*B Signaling Pathway in I/R-Induced Brain Injury

**Figure 6:**
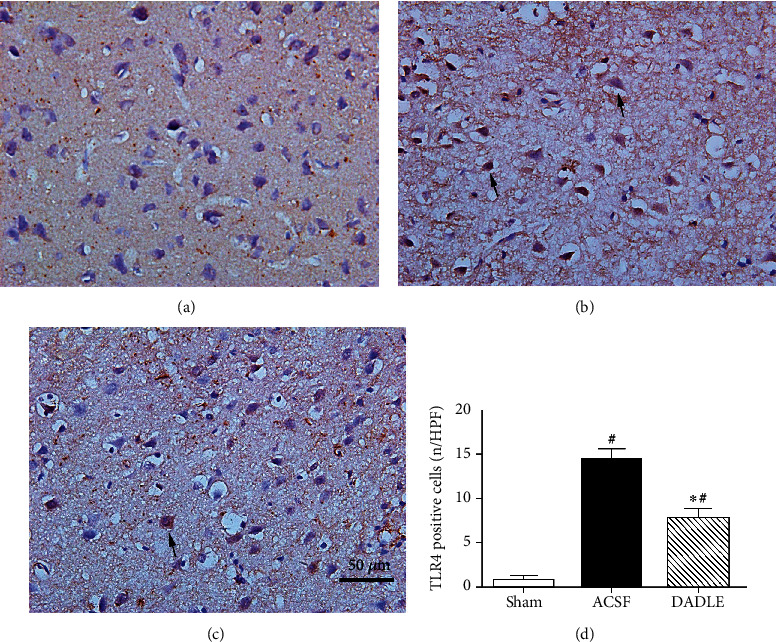
Immunohistochemical results of TLR4 expression in the ischemic penumbra at 24 hours after reperfusion. There was little TLR4 staining in the Sham group (a), while there was strong signal staining in the ACSF group (b), which was markedly reduced by DADLE (c) (original magnification ×400). The arrows indicate TLR4-positive cell. (d) Quantitative analysis of the number of TLR4-positive cells in the ischemic penumbra. Scale bar, 50 *μ*m; Bars represent mean ± SD (all groups *n* = 5). DADLE: [D-Ala^2^, D-Leu^5^]; ACSF: artificial cerebrospinal fluid. ^∗^*P* < 0.05 versus the ACSF group. ^#^*P* < 0.05 versus the Sham group.

**Figure 7:**
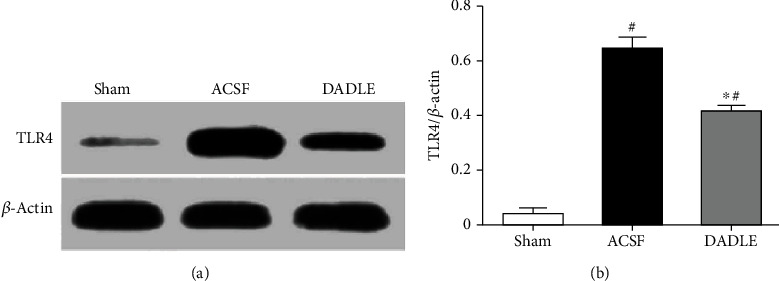
Effects of DADLE on the protein bands of TLR4 (a) expression in the ischemic penumbra at 24 hours after reperfusion. Quantitative analysis of TLR4 expression in (b). The expression of TLR4 proteins was up-regulated in the ACSF group and decreased after DADLE treatment. Bars represent mean ± SD (group DADLE *n* = 6, other groups *n* = 5). DADLE: [D-Ala^2^, D-Leu^5^]; ACSF: artificial cerebrospinal fluid. ^∗^*P* < 0.05 versus the ACSF group. ^#^*P* < 0.05 versus the Sham group.

It is well known that the TLR4/NF-*κ*B pathway is involved in the inflammatory reactions after brain insult. To further observe DADLE's effect on upstream inflammatory signaling in the brain of stroke models, we further examined the levels of TLR4 and the most classic transcription factor of NF-*κ*B. Few TLR4-immunopositive cells were found in the ischemic penumbra in sham-operated rats at 24 hours after reperfusion (Figure 6(a)), while the cells were obviously augmented in rats exposed to I/R treated with ACSF (Figure 6(b)). However, DADLE treatment remarkably decreased TLR4-immunopositive cells in the brain samples (Figure 6(c)). These results were also corresponded with the western blotting results of TLR4 protein expression. The level of TLR4 protein expression in the ACSF group had a significant increase to 19-fold of that in the Sham group at 24 hours after reperfusion, which was attenuated by DADLE adversely (45.9% of ACSF group, *P* < 0.05) (Figure 7).

**Figure 8:**
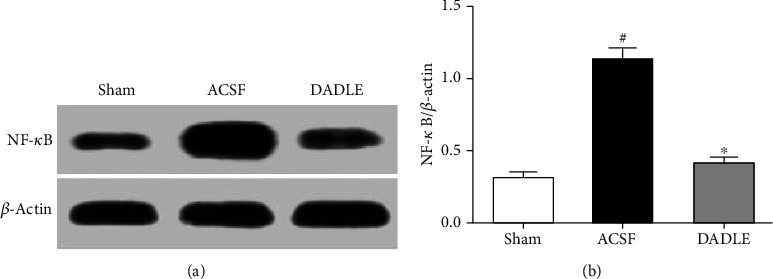
Effects of DADLE on the protein bands of NF-*κ*B p65 expression in the ischemic penumbra at 24 hours after reperfusion (a). Quantitative analysis of NF-*κ*B p65 expression in (b). The expression of NF-*κ*B p65 protein was upregulated in the ACSF group and decreased after DADLE treatment. Bars represent mean ± SD (group DADLE *n* = 6, other groups *n* = 5). DADLE: [D-Ala^2^, D-Leu^5^]; ACSF: artificial cerebrospinal fluid. ^∗^*P* < 0.05 versus the ACSF group. ^#^*P* < 0.05 versus the Sham group.

We also investigated NF-*κ*B activation by testing the protein level of the NF-*κ*B p65 subunit in the nucleus. A low level of NF-*κ*B p65 was found in ischemic penumbra in the Sham group. Compared with the Sham group, the expression of NF-*κ*B p65 was significantly increased, which was attenuated by DADLE administration (*P* < 0.05, Figures 8(a) and 8(b)).

The above results indicated that the cerebral protective mechanisms of DADLE were associated with the inhibition of TLR4/NF-*κ*B signaling pathway.

## 4. Discussion

Cerebral ischemia injury remains as a leading cause of neurological disability and mortality worldwide [1]. Thrombolysis as a stroke therapy was limited by the short therapeutic time window and risk of hemorrhage. Despite intensive efforts to develop neuroprotective methods in experimental cerebral ischemia, few have been translated to an effective therapeutic strategy in the clinic to date [21]. Therefore, understanding the underlying mechanism of drug action will promote the rational use of drugs for ischemic stroke in clinic.

DADLE, an analog of the endogenous delta-opioid enkephalin, attracted more attention as a link between hibernation and neuroprotection initially [22]. However, it was gradually found that DADLE could increase the threshold of tolerance to tissue ischemia. Our previous studies suggested that administration of DADLE could alleviate spinal cord I/R injury [3]. In this study, we demonstrated that intracerebroventricular injection of DADLE could significantly attenuate cerebral damage induced by I/R. These results were also consistent with previous studies involving several animal models of another organ I/R injury [23, 24].

In our study, the SD rat was subjected to 120 minutes of ischemia followed by 24 hours of reperfusion. The volume of cerebral infarction in our study was consistent with previous reports in rats treated with vehicle together with significant focal neurological deficits, indicating that the modeling was successful. No difference in mortality was found between the vehicle and DADLE group, but a significant difference was shown in cerebral infarction volume, suggesting a protective effect of DADLE on cerebral I/R injury.

As we all know, multiple mechanisms contribute to the progression of cerebral I/R injury, including oxidative stress, inflammation, energy failure, calcium overload, and glutamate excitotoxicity and apoptosis [6, 7]. Excessive inflammation is emerging as an important pathophysiological feature underlying the long-term neurological disorders. Proinflammatory reactions are triggered at the onset of cerebral I/R and then accelerate brain damage further. It was reported that the suppression of inflammatory presented a promising target for therapeutic intervention in ischemic brain damage [8]. DADLE was reported to ameliorate ischemic injury in rats by enhancing antioxidant ability and repressing caspase activity [11]. However, much of the research in organ protection related to DADLE focused on its antiapoptotic property without considering its potential effect on the inflammatory response. Thus, this study focused on the role of DADLE on inflammatory cytokines and signaling pathways. We firstly investigated the anti-inflammatory property of DADLE and then examined the potential mechanism underlying this action.

TNF-*α* and IL-6 are two key proinflammatory cytokines, which participate in the pathological process of cerebral I/R. In this study, we demonstrated that TNF-*α* and IL-6 were upregulated after transient MCAO, which was consistent with the previous study [25]. Meanwhile, we found that the upregulated levels of TNF-*α* and IL-6 were attenuated when treated with DADLE, which might be linked with neuroprotective features of DADLE.

After finding the anti-inflammatory effects of DADLE, we tried to explain the reason. TLR4 is the best-characterized pattern recognition receptor that can mediate the inflammatory signaling pathway and play an important role during ischemic damage in several organs [26–29]. A previous study reported that compared with wild-type mice, the TLR4 knockout mice subjected to cerebral I/R injury displayed smaller cerebral infarct size and neurological scores [14]. In this study, we observed an increase of TLR4 expression after ischemia accompanied with cerebral damage and neurologic retard, which were consistent with previous findings [30, 31]. Furthermore, our data proved that DADLE repressed I/R-induced TLR4 expression, which led to downregulation of cerebral inflammation after ischemic stroke.

We further investigated the mechanism through which TLR4 inhibited the inflammatory response. The transcription factor NF-*κ*B, a downstream molecule stimulated by TLR4, can modulate a vast number of genes' expression including cytokines, chemokines, and adhesion molecules [32]. It was reported that TLR4 expression was markedly upregulated in a rat model of global cerebral ischemia, which was accompanied with upregulated expression of NF-*κ*B and TNF-*α* [33]. Therefore, we hypothesized that DADLE might mediate neuroprotection through the TLR4/NF-*κ*B signaling pathway. Consequently, we found that DADLE downregulated the expression of NF-*κ*B p65 after transient focal cerebral ischemia, which suggested that DADLE could restrain the inflammatory reaction possibly via inhibiting TLR4/NF-*κ*B signaling pathway.

There are several limitations in the present study. Firstly, we only examined the influence of DADLE on TNF-*α*, IL-6, TLR4, and NF-*κ*B, however, which type of cell DADLE is acting on in the brain remains unknown. Thus, we will further investigate which specific cell the delta-opioid is acting on. Secondly, stereotactic injection lentivirus-mediated TLR4 overexpression should be used to verify the neuroprotective effect of DADLE. Moreover, our results were only found in experimental ischemic stroke. More clinical studies are needed to validate the benefits of DADLE based on extensive animal studies.

## 5. Conclusions

In conclusion, intracerebroventricular administration of DADLE can ameliorate cerebral I/R injury-induced neurological impairment and neuronal injury. The neuroprotective effect of DADLE was potentially through an anti-inflammatory mechanism involving suppression of the TLR4/NF-*κ*B signaling pathway. Therefore, our findings suggest that DADEL may be an effective intervention to attenuate brain damage to improve stroke outcome.

## Figures and Tables

**Table 1:** Physiological parameters.

Variables	Sham	ACSF	DADLE
Body weight (g)	227 ± 9	226 ± 10	225 ± 12
Rectal temperature (°C)			
Preischemia 5 minutes	37.3 ± 0.6	37.3 ± 0.8	37.4 ± 0.7
Ischemia 10 minutes	37.2 ± 0.8	37.1 ± 0.9	37.3 ± 0.9
Ischemia 30 minutes	37.3 ± 0.6	37.2 ± 0.8	37.2 ± 0.6
Ischemia 60 minutes	36.8 ± 0.8	37.0 ± 0.9	37.1 ± 0.8
MAP (mmHg)			
Preischemia 5 minutes	93 ± 9	98 ± 10	96 ± 8
Ischemia 10 minutes	96 ± 9	99 ± 7	97 ± 9
Ischemia 30 minutes	93 ± 6	97 ± 10	96 ± 8
Ischemia 60 minutes	90 ± 8	96 ± 9	92 ± 8

Physiological parameters were taken before ischemia and within 60 minutes following ischemia. No significant differences were observed in body weight, rectal temperature, and MAP among groups at different times (*P* > 0.05). Data are expressed as mean ± SD (*n* = 5). DADLE: [D-Ala^2^, D-Leu^5^]; ACSF: artificial cerebrospinal fluid; MAP: mean arterial pressure; 1 mmHg, 0.133 kPa.

## Data Availability

The data used to support the findings of this study are available from the corresponding author upon request.
